# Prepared to practice? Perception of career preparation and guidance of recent medical graduates at two campuses of a transnational medical school: a cross-sectional study

**DOI:** 10.1186/s12909-016-0584-6

**Published:** 2016-02-09

**Authors:** Sameer S. Kassim, Yvonne McGowan, Hannah McGee, David L. Whitford

**Affiliations:** Department of Family and Community Medicine, Royal College of Surgeons in Ireland-Bahrain, P.O. Box 15503, Adliya, Kingdom of Bahrain; Department of Medical Microbiology and Infectious Diseases, University of Manitoba, Room 543 Basic Medical Sciences Building, 745 Bannatyne Avenue, Winnipeg, MB R3E 0J9 Canada; Division of Population Sciences, Royal College of Surgeons in Ireland, 123 St Stephens Green, Dublin 2, Republic of Ireland; Faculty of Medicine and Health Sciences, Royal College of Surgeons in Ireland, 123 St Stephens Green, Dublin 2, Republic of Ireland

**Keywords:** Career preparation, Transnational medical education, Mentorship, International medical students, PHPQ

## Abstract

**Background:**

Graduating medical students enter the workforce with substantial medical knowledge and experience, yet little is known about how well they are prepared for the transition to medical practice in diverse settings. We set out to compare perceptions of medical school graduates’ career guidance with their perceptions of preparedness to practice as interns. We also set out to compare perceptions of preparedness for hospital practice between graduates from two transnational medical schools.

**Methods:**

This was a cross-sectional study. A Preparedness for Hospital Practice (PHPQ) survey and career guidance questionnaire was sent to recent medical graduates, incorporating additional free text responses on career preparation. Data was analyzed using descriptive statistics and tests of association including Chi-square, Mann-Whitney U and Kruskal-Wallis H tests.

**Results:**

Forty three percent (240/555) of graduates responded to the survey: 39 % of respondents were domestic (Dublin, Ireland or Manama, Kingdom of Bahrain) and interning locally; 15 % were overseas students interning locally; 42 % were overseas students interning internationally and 4 % had not started internship. Two variables explained 13 % of the variation in preparedness for hospital practice score: having planned postgraduate education prior to entering medical school and having helpful career guidance in medical school. Overseas graduates interning internationally were more likely to have planned their postgraduate career path prior to entering medical school. Dublin graduates found their career guidance more helpful than Bahrain counterparts. The most cited shortcomings were lack of structured career advice and lack of advice on the Irish and Bahraini postgraduate systems.

**Conclusions:**

This study has demonstrated that early consideration of postgraduate career preparation and helpful medical school career guidance has a strong association with perceptions of preparedness of medical graduates for hospital practice. In an era of increasing globalization of medical education, these findings can direct ongoing efforts to ensure all medical students receive career guidance and preparation for internship appropriate to their destination.

## Background

Graduating medical students enter the workforce with substantial medical knowledge and clinical experience, yet preparation and guidance for the transition to medical practice varies greatly across medical students and medical schools [1]. In one national study, the majority of trainees were dissatisfied with the career guidance provided by their institutions [2], and inadequate career preparation has been identified as one of the key stressors for medical students [1, 3, 4]. The reasons for this are most likely complex and will include an interaction between the inadequacy of institutional career guidance [5] and levels of awareness of the need for career preparation on the part of medical students, including the false perception that career guidance is complete once it has placed a person in medical school on a career trajectory towards being a doctor.

Within the Irish context, career guidance has been defined by the National Guidance Forum as “facilitating people throughout their lives to manage their own educational, training, occupational, personal, social, and life choices so that they reach their full potential and contribute to the development of a better society… The activities may take place on an individual or group basis, and may be face-to-face or at a distance.” [6] This definition and subsequent implementation in the medical school context incorporate several theories of guidance of relevance to medical students and challenge any perception that guidance in medical school is not essential. Firstly, the majority of medical students have a good degree of self-efficacy, but guidance can direct them into applying their self-efficacy more effectively in career choices, as advocated in Bandura’s social cognitive theory (self-efficacy resulting in reaching one’s goals) [7]. Additionally, Law’s theory of community interaction (career choice occurring in the context of social community interaction) [8] would advocate utilising individual experiences and peer influences within medical school and clinical attachments to guide career choices. Furthermore, medical school is a time of major life transition for most individuals and both Holland’s theory of vocational types (occupational choice as an expression of personality) [9] and Super’s developmental self-concept theory (vocational development related to development of self-concept over life-span) [10] are useful in enabling medical students to reflect on their own self and their life transitions and make choices based around these. The value of career guidance becomes most acute for the medical student on completion of medical school training and entrance into working life as an intern.

Career guidance throughout medical school is therefore important in encouraging these developments within an individual and assisting the individual to reflect on their own personality, experiences, life stage and personal goals in order to make career choices. The majority of careers counsellors within medical schools would utilise these theories and a similar definition in order to facilitate career choices of medical students. Conclusive evidence of the outcomes of career guidance continue to be beset by a recurring set of issues, particularly the lack of agreed outcome measures [11, 12]. However, evidence exists that career guidance promotes attitudinal change and shifts in motivation [11, 12], and has economically relevant benefits for individuals by supporting smooth and rapid transitions in learning and work [13, 14]. In a similar way, we would hypothesise that good career guidance within medical school would lead to an improved transition between medical school and internship i.e. feel better prepared for hospital practice.

An additional factor contributing towards the challenge of career guidance in medical schools is the increasing globalisation of medical education. International and transnational medical schools have been shown to have a considerable impact on the supply of medical practitioners for medical workforces [15, 16]. International mobility in the medical workforce for both education and careers means that good career preparation and guidance is even more necessary to ensure that graduates are adequately prepared for postgraduate training across different territories. The Royal College of Surgeons in Ireland (RCSI) is a transnational educator having undergraduate medical schools in three regions (Europe, Middle East and Far East) and serving students from over 60 different countries.

In this study, we set out to compare perceptions of RCSI medical school graduates’ career guidance with their perceptions of preparedness to practice as interns. We also set out to compare perceptions of preparedness for hospital practice between graduates from two of the transnational campuses of the RCSI.

## Methods

The Royal College of Surgeons in Ireland has been providing undergraduate medical education since 1886. RCSI operates the largest undergraduate medical school in Ireland with an annual enrolment of more than 300 students distributed through 4 year (Graduate Entry Medicine (GEM)), 5 year and 6 year medical programs. Since 2004, RCSI also operates the Medical University of Bahrain (RCSI Bahrain) in the Kingdom of Bahrain, a constituent university within the RCSI system. It has an annual medical school enrolment of over 140 students into the 5 year and 6 year medical programs.

We carried out a cross-sectional study using an online questionnaire. All RCSI medical graduates from Dublin and Bahrain in 2010 and 2011 were included in this study. Email addresses of the graduates were obtained from the alumni offices of both campuses. A link to an online questionnaire utilizing Survey Monkey® and incorporating the Preparedness for Hospital Practice Questionnaire (PHPQ) [17, 18] and a survey regarding career preparation was emailed to each graduate from the alumni offices in both Dublin and Bahrain. Two reminders were sent from the alumni offices with a final third reminder from the University CEO in order to increase the response rate.

The PHPQ is a valid and reliable 41-item questionnaire which contains eight subscales designed to assess key areas of medical hospital practice:

1. Interpersonal skills: effective and competent communication with hospital patients.
2. Confidence/coping skills.
3. Collaboration: the importance of a team approach to care.
4. Practical skills and patient management.
5. Understanding science: understanding of the scientific basis of disease and therapeutics.
6. Prevention: preparedness to incorporate health promotion and disease prevention into hospital practice.
7. Holistic care: an appreciation of the impact of multiple variables on a patient’s health and disease.
8. Self-directed learning: evaluation of performance, identification of educational needs and extension of knowledge and skills.

We used a version of the PHPQ questionnaire with a 5-point Likert scale from very inadequately prepared to very adequately prepared as previously published [19], instead of the 6-point scale as originally published [17]. Responses to career preparation were scored on a 3-point scale from unhelpful to helpful. Three free text response questions were also used within the survey to gather data regarding career preparation. The first two questions addressed perceived strengths and weaknesses of the career guidance in medical school with a third question seeking suggestions for improvement of careers guidance.

Statistical analysis was carried out using Statistical Package for the Social Sciences (SPSS 21.0 for Windows). Following descriptive statistics, associations between characteristics were established using chi-squared, Mann-Whitney U and Kruskal Wallis tests of association. The Framework Method of thematic analysis was used to analyse the free text responses as it enabled us to compare and contrast data by themes across many graduates, while also retaining the connection to other aspects of each graduate’s account [20].

This study was approved by the Research Ethics Committees of both RCSI Dublin and RCSI Bahrain. Information on the study was given to participants. Consent to participate was given through return of the completed questionnaire.

## Results

### Demographics

The response rate to the survey was 43 % (240/555). Three groups of graduates were identified according to their home origin and their internship location and campus. Thirty nine percent (93/240) were ‘*domestic graduates and interning locally’* in Ireland or Bahrain; 15 % (37/240) were ‘*overseas graduates interning locally’* in Ireland or Bahrain; and 42 % (100/240) were ‘*overseas graduates interning internationally’*. Four percent of graduates at the time of survey had not started internship or did not complete this section and were excluded from analysis. The relationship between these three groups and the characteristics of the respondents are shown in Table 1. The three groups differed in age, length of medical program, nationality, internship placement, career preference and planning for postgraduate education.

**Table 1:** Characteristics of respondents with comparison between internship groups and campus of graduation

Characteristic		Internship Group			Statistic	Campus of Graduation		Statistic
		Domestic students interning locally (*n* = 94)	Overseas student interning locally (*n* = 37)	Overseas student interning internationally (*n* = 100)		RCSI Dublin graduates (*n* = 187)	RCSI Bahrain graduates(*n* = 53)	
Age		27.5 + 3.6	25.9 + 2.4†	27.2 + 2.4	*F* = 4.3, 2df, *p* = 0.01	27.3 + 2.98	25.2 + 2.01	*F* = 31.1, 1df, *p* < 0.01
Gender	Male	41 (43.6 %)	17 (45.9 %)	52 (52 %)	Χ^2^ = 1.3, 2df, *p* = 0.53	92 (49.2 %)	25 (52.8 %)	*P* = 0.07, (two sided, Fisher’s exact test)
	Female	53 (56.4 %)	20 (54.1 %)	48 (48 %)		95 (50.8 %)	28 (47.2 %)	
Program	4	32 (34 %)	2 (5.4 %)	24 (24 %)	Χ^2^ = 18.4, 4df, *p* < 0.01	59 (31.6 %)	0 (0.0 %)	Χ^2^ = 27.7, 2df, *p* < 0.01
	5	34 (36.2 %)	23 (62.2 %)	32 (32 %)		73 (39.0 %)	21 (39.6 %)	
	6	28 (29.8 %)	12 (35.7 %)	44 (44 %)		55 (29.4 %)	32 (60.4 %)	
Planned for Post Graduate Medical Education	Yes	27 (31 %)	20 (55.6 %)	84 (87.5 %)	Χ^2^ = 3.2 3df, *p* = 0.37	103 (57.9 %)	37 (72.5 %)	*P* = 0.07, (two sided, Fisher’s exact test)
Nationality	Europe (Ireland)	68 (73.1 %)	0	0	Χ^2^ = 222.4, 10df, *p* < 0.01	68 (36.4 %)	0 (0.0 %)	Χ^2^ = 73.8, 6df, *p* < 0.001
	Europe (Other)	0	5 (13.5 %)	10 (10 %)		12 (6.4 %)	5 (9.4 %)	
	Middle East	25 (26.8 %)	4 (10.8 %)	19 (19 %)		17 (9.1 %)	30 (56.6 %)	
	North America	0	7 (18.9 %)	56 (56 %)		61 (32.6 %)	9 (17.0 %)	
	Africa	0	3 (8.1 %)	3 (3 %)		3 (1.6 %)	3 (5.7 %)	
	Asia/Pacific	0	15 (40.5 %)	10 (10 %)		21(11.2 %)	1 (1.9 %)	
	No response	0	3 (8.1 %)	2 (2 %)		0 (0.0 %	0 (0.0 %)	
Internship Region	Europe (Ireland)	69 (74.2 %)	30 (81.1 %)	0 (0.0 %	Χ^2^ = 186.2, 10df, *p* < 0.01	99 (52.9 %)	0 (0.0 %	Χ^2^ = 129.0, 6 df, *p* < 0.01
	Europe (Other)	0	0 (0.0 %	14 (14 %)		10 (5.4 %)	4 (7.5 %)	
	Middle East	24 (25.8 %)	7 (18.9 %)	17 (17 %)		19 (10.2 %)	34 (64.2 %)	
	North America	0	0	63 (63 %)		57 (30.5 %)	6 (11.3 %)	
	Other	0	0	1 (1.0 %)		2 (1 %)	9 (17.0 %)	
	No response	1 (1.1 %)		5 (5.0 %)		0 (0.0 %)	0 (0.0 %)	
Career Preference	General Practice	21 (22.6 %)	4 (10.8 %)	21 (21 %)	Χ^2^ = 20.7, 8df, *p* < 0.01	39 (20.5 %)	8 (15.1 %)	Χ^2^ = 2.3, 5df, *p* = 0.81
	Medicine and sub-specialties	26 (28 %)	6 (16.2 %)	33 (33 %)		50 (26.7 %)	18 (34.0 %)	
	Surgery and sub-specialities^a^	14 (15.1 %)	9 (24.3 %)	25 (25 %)		38 (20.3)	12 (22.6 %)	
	Pediatrics	8 (8.6 %)	10 (27 %)	6 (6 %)		19 (10.2 %)	6 (11.3 %)	
	Other^b^	23 (24.7 %)	8 (21.6 %)	15 (15 %)		40 (21.4 %)	9 (17.0 %	

^a^Includes: Emergency Medicine

^b^Includes: Anaesthesiology, Dermatology Lab Medicine, Obs/Gyn, Ophthalmology, Psychiatry, Radiology, Research/Education

### Perception of career preparedness and guidance

Thirty nine percent (94/232) of respondents rated their career guidance as helpful, whilst 28 % (67/232) regarded it as unhelpful. Dublin graduates were more likely to find their career guidance helpful than Bahrain graduates (46 % v. 22 %, χ^2^ = 10.8, df 2, *p* < 0.01). Graduates were asked to indicate their career preference following graduation. The most cited specialties were medicine (28 %), surgery (21 %) and general practice (20 %). There was no overall difference in satisfaction with career guidance and preparation between specialties chosen. No differences in satisfaction with career guidance and preparation were noted across gender, nationality, program duration (4/5/6 years) or year of graduation. Overseas graduates interning internationally were more likely to have planned their postgraduate career path prior to entering the medical school compared to their domestic or overseas local counterparts (87.5 % v. 31 %, χ^2^ = 60.8, df 2, *p* < 0.01).

### Preparedness for Hospital Practice Questionnaire (PHPQ)

Scores for the domains of the PHPQ are shown in Table 2. There was no association between the domains of the PHPQ and gender, year of graduation, medical school site and career preference.

**Table 2:** Preparedness for Hospital Practice Questionnaire Domain Scores with comparison between internship groups and campus of graduation

Domain	Total		By Internship Group						By Campus of Graduation			
			Domestic students interning locally (*n* = 93)		Overseas student interning locally (*n* = 37)		Overseas student interning internationally (*n* = 100)		RCSI Dublin graduates (*n* = 187)		RCSI Bahrain graduates (*n* = 53)	
	Mean	95 % CI^a^	Mean	95 % CI^a^	Mean	95 % CI^a^	Mean	95 % CI^a^	Mean	95 % CI^a^	Mean	95 % CI^a^
Interpersonal skills	3.44	3.33–3.56	3.29	3.11–3.48	3.35	3.03–3.68	3.64 ^*^	3.47–3.80	3.52	3.39–3.64	3.17 ^**^	2.89–3.45
Confidence	3.55	3.46–3.65	3.36	3.20–3.51	3.39	3.13–3.66	3.80 ^*^	3.67–3.94	3.52	3.42–3.63	3.66	3.43–3.88
Collaboration	3.52	3.41–3.62	3.43	3.26–3.60	3.48	3.19–3.77	3.63	3.47–3.78	3.51	3.39–3.62	3.56	3.34–3.77
Patient management	3.48	3.40–3.57	3.50	3.37–3.63	3.41	3.15–3.67	3.52	3.39–3.65	3.52	3.42–3.61	3.37	3.16–3.57
Understanding science	3.64	3.55–3.73	3.63	3.48–3.77	3.46	3.19–3.72	3.73	3.58–3.88	3.67	3.57–3.77	3.53	3.31–3.76
Prevention	3.96	3.88–4.04	3.88	3.75–4.01	3.86	3.64–4.08	4.09 ^*^	3.99–4.20	3.95	3.86–4.04	4.01	3.84–4.17
Holistic care	3.92	3.84–4.01	3.74	3.60–3.88	3.73	3.50–3.95	4.17 ^*^	4.06–4.29	3.87	3.78–3.97	4.11^**^	3.90–4.31
Self-directed learning	3.93	3.84–4.01	3.90	3.77–4.03	3.72	3.46–3.97	4.06 ^*^	3.94–4.18	3.92	3.83–4.01	3.95	3.74–4.16

^a^CI = confidence interval

Kruskal-Wallis, ^*^ = *p* < 0.05

Mann-Whitney U, ^**^ = *p* < 0.05

Graduates of Irish nationality scored lower in the domains of confidence, holistic care and self-directed learning (Kruskal-Wallis H, χ^2^ = 21.71, 27.64, 11.53, df = 4, *p* < 0.001, 0.001, 0.021) as compared with graduates from North America, other parts of Europe and the Middle East. RCSI Dublin graduates felt more prepared for interpersonal relationships with patients, but less well prepared to provide holistic care compared to their Bahrain based counterparts (U = 3438, 3451, *p* = 0.023, 0.025).

Given the transnational context of RCSI, graduates were grouped according to their origin and internship destination. In this analysis, international graduates interning internationally scored higher in the domains of interpersonal skills (Kruskal-Wallis H, χ^2^ = 6.2, *p* = 0.045), confidence (Kruskal-Wallis H, χ^2^ = 19.12, *p* < 0.001), prevention (Kruskal-Wallis H, χ^2^ = 6.06, *p* = 0.048), holistic care (Kruskal-Wallis H, χ^2^ = 25.30, *p* < 0.001) and self-directed learning (Kruskal-Wallis H, χ^2^ = 10.29, *p* = 0.006) than domestic graduates interning locally (Table 2).

**Fig. 1:**
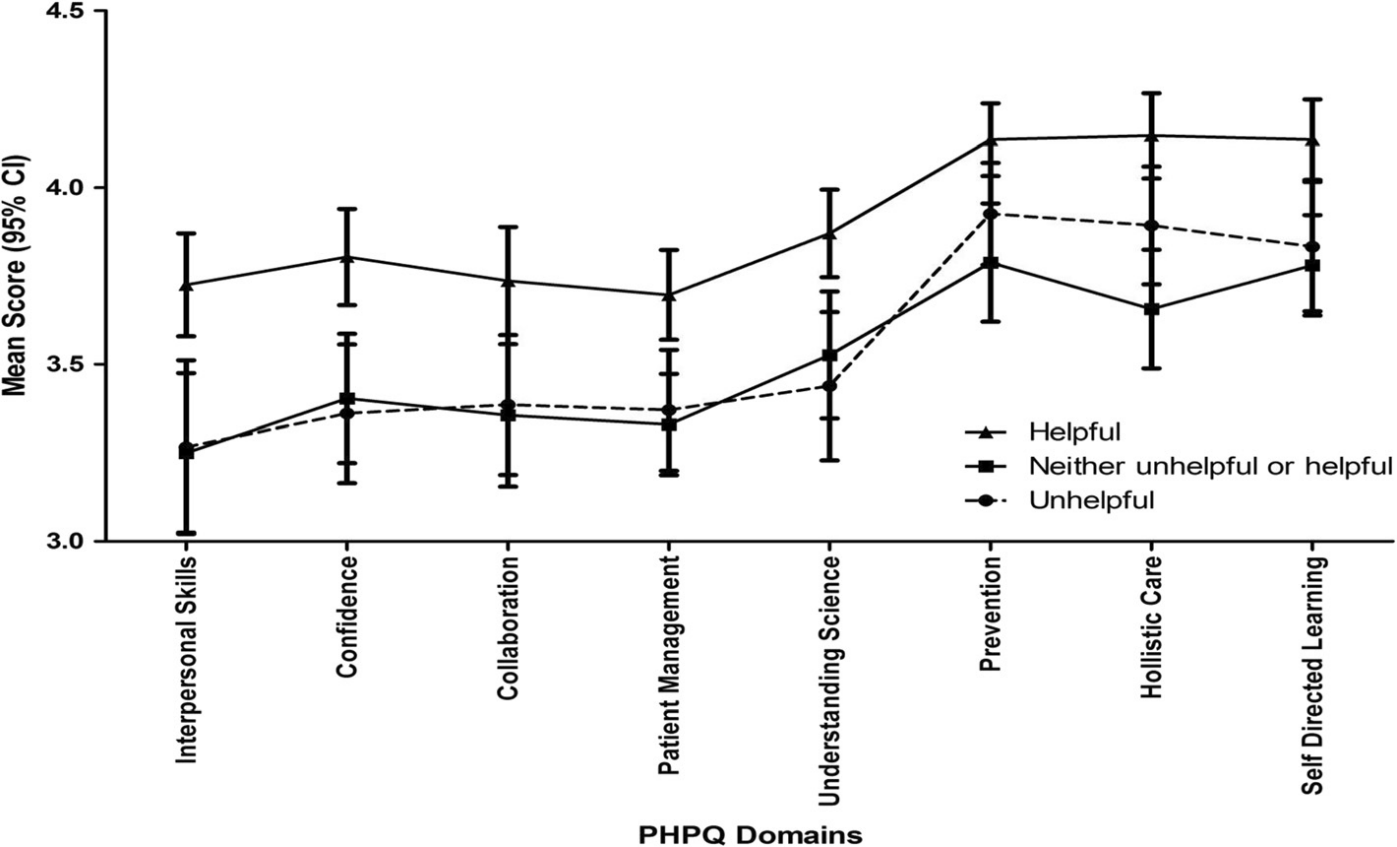
Preparedness for Hospital Practice Domain Scores as related to Helpfulness of Medical School Career Guidance

**Fig. 2:**
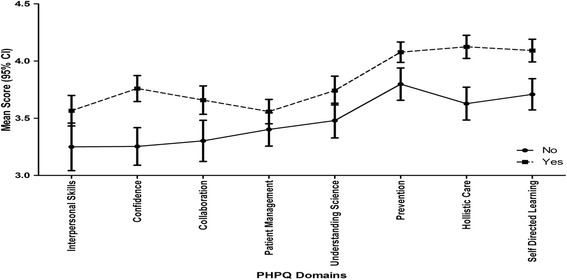
Preparedness for Hospital Practice Domain Scores as related to Planning for Postgraduate Medical Education prior to entering medical school

Graduates assessing their career guidance as helpful scored higher across most domains of the PHPQ as compared with graduates assessing their career guidance as either unhelpful or neither helpful nor unhelpful (Fig. 1). Graduates who had planned for their postgraduate medical education prior to entering medical school scored higher across most domains of the PHPQ as compared with graduates who had not prepared (Fig. 2). Linear regression analysis indicated that two variables explained 13 % of the variation in preparedness for hospital practice score: planning postgraduate education prior to entering medical school (t = 4.2, *p* < 0.01); and having helpful career guidance in medical school (t = 3.9, *p* < 0.01).

### Qualitative comments

Graduates commented most favorably on career guidance from external lecturers, workshops organized by student societies and ad hoc discussions with consultants. The areas most negatively commented on were the deficiency of structured advice on surgical training and the lack of guidance related to the Irish and Bahraini postgraduate training systems.

Graduates reported they would have liked more exposure to sub-specialties, time to explore non-clinical options and exposure to health systems outside of North America, Ireland and the Middle East. Furthermore, one of the most frequent suggestions from recent graduates was for the delivery of formal lectures in career guidance starting as early as pre-clerkship. Graduates suggested that formal career mentorship from a qualified individual in the field would also be beneficial for career planning. Graduates felt that a careers office to coordinate electives, give access to information regarding career pathways and to advise on training requirements for new graduates would be beneficial to their advancement in medical education (Table 3).

**Table 3:** Selected comments from graduates regarding career preparation at RCSI Dublin and RCSI Bahrain

	RCSI Dublin	RCSI Bahrain
What was done well	• Kaplan and special lectures geared towards USMLEs and the US application process was invaluable • Events organised by the Surgical Society, wherein consultant surgeons from various hospitals presented an overview of their careers and provided advice for those aspiring to be surgeons • Careers day with speakers from number of different countries • RCSI alumni and senior students from North America advising based on their own experience	• Kaplan and special lectures towards USMLE
What could use improvement	• I cannot recall formal career advice regarding careers in Ireland. There was advice available for the North Americans or for Irish people wishing to pursue residency in the US • I received little to no personal guidance on which specialty would best serve my interests and goals. My choice was based almost entirely on my clinical experience. I would have liked to have had a mentor whom I could have met with a few times 1:1 • Discussion on career paths within the Irish system. Advice on alternatives to clinical practice.	• We did not have proper guidance during our medical education • If someone wanted to take a career path that did not involve practicing in a hospital, there was no one or nowhere to go to for help. Students need to be aware of ALL their choices and should be offered help concerning where they want to go • Seminars on the exact requirements to join any program in each country outside North America and Ireland • Would be helpful if Alumni were available to speak to the students to help guide them on their path and give any advice based on their experiences
What additional resources would recent graduates prefer in their learning environments	• Career guidance day, where PRACTICAL information regarding the different medical and surgical specialities are discussed. Such practical information remains quite difficult to obtain and mystified when training in medical school • Specific career guidance talks, seminars on training paths & research needed for various specialties. Diagrams with training paths early on in medical school would have helped us understand what lay ahead • Assigning career mentors at junior levels who can provide advice about career planning • Clinical facilitator to ensure students have access to speciality and subspecialty electives locally and abroad	• Career counsellor should be at our university and should meet with each student • A staff person to help arranging overseas electives and guidance as well as assist with regarding options available after graduation

## Discussion

This study has demonstrated that early consideration of postgraduate career preparation and helpful medical school career guidance has a strong association with perceived preparedness for hospital practice of medical graduates. This is in line with research from other fields showing that career guidance supports smooth and rapid transitions in learning and work [13, 14]. This study also showed that those attending a transnational medical school from overseas and returning overseas were more prepared for their future career as compared to their domestic counterparts.

We were able to quantify that medical graduates from overseas were more likely to have planned for their postgraduate medical education prior to entry to the College. It has been argued that the personal attributes of graduates such as patience, interest, motivation, being proactive, ambitious, and dedicated are more influential in achieving career goals and accessing further training than perceived features of any medical program [4]. This may be reflected in the international medical graduates surveyed here. Alternatively, it may reflect the greater maturity and experience of those entering medical school from overseas, with a greater proportion of overseas graduates already holding degrees.

Our study also indicates that the majority of graduates complete their early postgraduate training in their country of origin. A recent study from Australia showed that a student recruitment process that favoured those with interest in rural and remote interests delivered 88 % of its graduates to their intended communities compared to other universities in Australia [21]. This finding suggest that many medical school entrants ultimately return to their home communities, and international medical schools therefore need to prepare their graduates for this.

Our study has shown that RCSI is well suited to providing career planning and guidance to overseas students to allow them to return to their home countries. However, graduates completing internships within Ireland and Bahrain were less likely to be satisfied with their undergraduate career advice and preparation. This highlights some key issues applicable to ‘local’ students. Local students may be less prepared than overseas students for career planning. In addition, it is likely that assumptions are made by both individuals and the institution that career development is more straightforward for local students. These assumptions need challenging and earlier and more structured medical career planning for students needs to be provided.

This is the first study to compare perceptions of career preparedness of new medical graduates of a transnational medical school. Of particular interest to other transnational and international medical schools is the maintenance of similar standards across campuses in different countries. It is of note that there was little difference between perceptions of preparedness for hospital practice between Ireland and Bahrain graduates, particularly in the light of significant differences in the level of preparedness between medical schools within the United Kingdom [3]. This suggests that shared objectives and learning outcomes between the campuses within a single institution are delivering similar outcomes, even in differing healthcare systems. However, we have shown that less than half of recent graduates were satisfied with the career guidance and preparation they had received, and that Bahrain graduates were less likely to be satisfied than those in Ireland. This may be related to several factors including the more recent establishment of the Bahrain campus and the less formalised system of hospital postgraduate training in the Kingdom of Bahrain. Bearing in mind that career satisfaction is associated with good career preparation, these issues need to be further elucidated and addressed.

One weakness of our study is the lack of information regarding pre-medical preparation and experiences of our enrolees. Furthermore, given that our response rate was only 43 % and the number of new graduates from RCSI Bahrain was limited in the first two years of graduation, the number of graduates that could be assigned to groups for comparison was relatively small. Given that the data showing significant differences between RCSI Dublin and Bahrain in terms of PHPQ domains exhibit larger confidence intervals, we would suggest caution in drawing statistical inferences from this data. In addition, it is noted that there was a significant difference between enrollees in Bahrain and Ireland that may explain some differences. Nevertheless, the results of this study are representative of new graduate experiences within our institution. These findings should however be evaluated more broadly within our own institution as well as in and between other transnational and international medical schools. The strength of this study is in elucidating perceptions of career guidance and preparedness for hospital practice from a diverse group of international medical graduates based on one institution in both a quantitative and qualitative manner. Good career advice and planning leads to improved satisfaction and outcomes later in a medical graduate’s career [2]. Since this study, RCSI has introduced a formalised careers management service which will provide an opportunity to evaluate change in graduate perceptions of preparedness for practice, and career advice satisfaction in the coming years.

## Conclusions

This study has highlighted the need to enhance early medical career planning and guidance for domestic as well as international career options for medical schools globally in order to facilitate the transition between medical school and internship. Given the rapidly changing world of medical education, from a country-specific model to one of international and transnational schools, the career preparation needs of students from diverse backgrounds need to be factored into the support systems of medical schools. The need to strengthen career planning and guidance not only within RCSI but within the broader context of the European and Middle East system of medical education should be a key focus for medical school directors. Further research needs to be done to address the needs of future students both local and international in order to more appropriately prepare this workforce for the needs of health services around the world.
